# Trait mindfulness is primarily associated with depression and not with fatigue in multiple sclerosis (MS): implications for mindfulness-based interventions

**DOI:** 10.1186/s12883-021-02120-z

**Published:** 2021-03-16

**Authors:** Torsten Sauder, Philipp M. Keune, Roy Müller, Thomas Schenk, Patrick Oschmann, Sascha Hansen

**Affiliations:** 1 Department of Neurology, Klinikum Bayreuth GmbH, Bayreuth, Germany; 2 Department of Psychology, Ludwig-Maximilians-University of Munich, Munich, Germany; 3 Department of Physiological Psychology, University of Bamberg, Bamberg, Germany

**Keywords:** Multiple sclerosis, Trait mindfulness, Depression, Fatigue, Mediation

## Abstract

**Objectives:**

Persons with MS (PwMS) often display symptoms of depression and fatigue. Mindfulness-based interventions are known to counteract these symptoms. However, to-date the exact relations between trait mindfulness, depression and fatigue remain to be examined. Fatigue is generally regarded as a symptom immanent to the disease and as a direct neurobiological consequence of increased cytokine levels and cortical atrophy. In depression on the other hand, psychosocial factors in the context of adaptation difficulties are probably of higher relevance. Hence, one may argue that mindfulness, as a trait that promotes successful adaption, may show a strong negative association with depression and a relatively minor negative association with fatigue in PwMS.

**Methods:**

In the current study, the association between self-reported trait mindfulness, fatigue and depression was examined in a sample of 69 PwMS.

**Results:**

Trait mindfulness showed highly significant negative correlations with both, depression and fatigue. Mediation analyses however, revealed that depression mediated the relation between mindfulness and fatigue.

**Conclusion:**

It may be concluded that in PwMS, trait mindfulness shows a genuine negative association with depression, but that it is only secondarily associated with fatigue. Implications for mindfulness-based interventions in MS are discussed. Based on the results of the current study, it may be feasible to promote the acceptance of default fatigue symptoms, instead of an actual reduction of fatigue symptoms.

## Introduction

Multiple Sclerosis (MS) is a chronic disabling neurological disease, leading to axonal degeneration and cortical atrophy [1]. Besides motor [2–4] and cognitive dysfunction [5–9] approximately 30% of persons with MS (PwMS) suffer from depression [10] and at least 75% from fatigue [11]. Fatigue has been defined as a “decrease in physical and/or mental performance that results from changes in central, psychological, and/or peripheral factors” [12]. In order to counteract these symptoms, mindfulness-based interventions (MBIs) have received considerable attention and have been successfully applied to PwMS in previous studies [13–15]. Mindfulness has been defined as paying attention to the present moment in a purposeful and nonjudgmental manner [16]. It can be conceptualized in terms of a *state* or in terms of a *trait* [17]. A mindful state is commonly achieved during the practice of mindfulness mediation. Trait mindfulness on the other hand has been described as a disposition of being mindful during activities of everyday life [17]. It has been shown that MBIs, i.e. repeated practice of state mindfulness, increase trait mindfulness over time and promote psychological health [18–22]. 

However, to-date the exact relation between trait mindfulness, depression and fatigue remains to be examined in PwMS. A recent meta-analysis indicated that MBIs may relieve fatigue in PwMS [23]. However, it may be possible, that this reduction of fatigue due to MBIs is a side effect of the primary reduction of depression. Compatible with that assumption, Mohr et al. [24] reported that the reduction of fatigue was primarily related to changes of depressive symptoms following treatment with psychotherapy or an antidepressant. The same might be assumed for MBIs which - as a third wave behavioral and cognitive therapy - also target depressive symptoms. 

In PwMS, fatigue is generally regarded as a disease-related symptom due to neurobiological alterations involving increased cytokine levels and axonal damage [25–27]. For depression, on the other hand, psychosocial factors might be of higher relevance than for fatigue. The diagnosis of a chronic and unpredictable disease like MS is often associated with feelings of uncertainty, hopelessness and the adoption of maladaptive coping strategies [28]. It is suggested that 40% of the variance in depression scores can be attributed to psychosocial factors such as uncertainty and emotion-focused coping in PwMS [29]. Hence, mindfulness as a trait that is associated with constructive coping [30] may promote successful adaptation. Since depression in MS is at least partly the result of maladaptive coping, trait mindfulness may show a strong negative association with depression and a relatively minor negative association with fatigue, as fatigue may be regarded as disease immanent. 

Fatigue is suggested to be a multidimensional construct incorporating physical and cognitive aspects. It has been proposed that physical fatigue is closely associated with disease-related factors, while cognitive fatigue might be more associated with psychosocial factors [31]. Therefore, it may be feasible to consider cognitive fatigue and physical fatigue separately. If cognitive fatigue is more related to psychosocial factors, it might be more susceptible to change induced by mindfulness than physical fatigue. 

The purpose of the current cross-sectional study was to examine the relation between trait mindfulness, depression, and fatigue in PwMS in more detail. Specifically, we hypothesized that depression may function as a mediator between mindfulness and fatigue. The latter constellation would suggest that the known beneficial effect of mindfulness on fatigue might in fact be attributable to a beneficial effect of mindfulness on depression. This in turn may have important clinical implications. If depression mediates the relation between trait mindfulness and fatigue, it may be suggested that future MBIs ought to promote salutatory adaptation to default fatigue symptoms instead of intending to reduce them.

## Methods

### Participants and procedure

The current study was approved by the ethics committee of the University of Bamberg, Germany. Participants were recruited sequentially in the outpatient clinic of the Department of Neurology, Klinikum Bayreuth GmbH, Germany. Patients were contacted by a scientific assistant during their waiting time in the anteroom of the outpatient clinic. To participate, patients needed to fulfill a diagnosis of MS based on the revised McDonald criteria [32] and to be at least 18 years old. Patients were only included if they were ambulatory without assistance for at least 500 m, indicated by an EDSS score of four or less, and had no other neurological diseases except MS. All patients who fulfilled these criteria were included in the study regardless of their level of depression or fatigue. Informed consent was obtained prior to participation. If patients were willing to participate, they completed several self-report measures addressing trait mindfulness, depression, and fatigue. Further, they completed several motor tasks referring to a different research question, results of which are to be reported elsewhere. In addition, demographic and clinical data were obtained from their files. In total *N* = 69 patients participated and were included in the analyses. Demographic and clinical characteristics of these patients are depicted in Table 1.

**Table 1:** Sample description

Demographics	Statistic
N (male/female)	18/51
Age (*M*, *SD*)	40.10, 10.85
Clinical characteristics	
MS type (N: RRMS, SPMS, PPMS)	69, 0, 0
Disability level (EDSS: median, range)	2, 0–4
Years since diagnosis (*M*, *SD*)	7.07, 5.97
Disease modifying therapy (N: yes, no)	59, 10
Self-report measures	
Depression (CES-D; *M*, *SD*)	13.46, 9.46
Depression (CES-D; N: yes, no)^a^	11, 58
Physical fatigue (WEIMuS; *M*, *SD*)	11.41, 8.72
Physical fatigue (N: yes, no)^a^	23, 46
Cognitive fatigue (WEIMuS; *M*, *SD*)	10.28, 8.91
Cognitive fatigue (N: yes, no)^a^	22, 47
Mindfulness (FMI; *M*, *SD*)	24.86, 6.44

*CES-D* Center for Epidemiological Studies Depression Scale, *EDSS* Expanded Disability Status Scale, *FMI* Freiburg Mindfulness Inventory, *M* mean, *PPMS* primary progressive MS, *RRMS* relapsing remitting MS, *SD* standard deviation, *SPMS* secondary progressive MS, *WEIMuS* Wuerzburger Fatigue Inventory for Multiple Sclerosis; ^a^presence of clinically relevant depressive symptoms and fatigue based on respective cut-off scores of the CES-D and WEIMuS, see self-report measures for details

### Self-report measures

In order to assess depressive symptoms, the German version of the Center for Epidemiological Studies Depression Scale (CES-D [33, 34]) was administered. The CES-D is an instrument used to quantify impairments due to depressive symptoms in the last week. It contains 20 items, which are ranked on a scale from zero to three (range: 0 to 60), and inquires emotional, motivational, cognitive, physical, and motor symptoms. A sum score of ≥24 is defined as the cut-off for clinically relevant depressive symptoms in the German version of the CES-D.

The Wuerzburger Fatigue Inventory for Multiple Sclerosis (WEIMuS [35]) is a self-assessment instrument tailored to PwMS to measure subjective fatigue in the last week. The 17 items are rated on a scale from zero to four yielding a general sum score (range: 0–68), and one for each of the two subscales physical (0–36) and cognitive fatigue (0–32). A higher score indicates a higher level of subjective fatigue. Cut-off scores for clinically relevant physical (≥16), cognitive (≥17) and general fatigue symptoms (≥32) were implemented [36].

The German short version of the Freiburg Mindfulness Inventory (FMI [37]) was implemented to assess trait mindfulness. It contains 14 items, which are ranked on a scale from one to four, yielding a sum score. The maximum score is 56 with a higher score indicating a higher level of trait mindfulness. The items reflect the basic components of mindfulness, i.e. presence and acceptance.

### Statistical analyses

To obtain an overview of the relations between the variables of interest, descriptive intercorrelations between trait mindfulness, depression, cognitive and physical fatigue were computed by means of Pearson correlations. Subsequently, two mediation analyses were implemented to analyze whether trait mindfulness predicted cognitive and physical fatigue and whether the respective association between mindfulness and fatigue was mediated by depression.

**Fig. 1:**
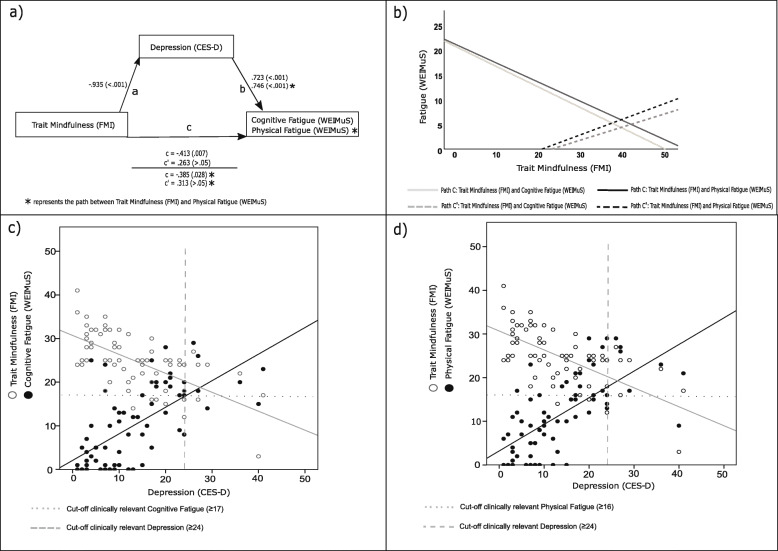
**a**) Schematic overview of the assumed mediation model. Results of the regression analyses are depicted as unstandardized B coefficients with respective p-values in parenthesis for each path (path c = total effect of trait mindfulness on fatigue, path c´ = direct effect while controlling for depression); **b**) Displays the respective regression lines of path c and path c´; **c** and **d** represent scatterplots of trait mindfulness and cognitive/physical fatigue and depression with dotted and dashed lines illustrating the cut-off scores of the respective self-report measures for clinically relevant depression and fatigue; *CES-D* Center for Epidemiological Studies Depression Scale, *FMI* Freiburg Mindfulness Inventory, *WEIMuS* Wuerzburger Fatigue Inventory for Multiple Sclerosis

Originally, Baron and Kenny [38] identified four steps required for meeting the criteria of mediation. More recently, it was called into question if all four steps are required as previously suggested by the authors [39]. Nevertheless, for a comprehensive illustration of the results and the relationships between the variables, in the current analysis all four steps were performed and are presented in Fig. 1a.

In the first step it was tested whether the assumed causal variable (trait mindfulness) predicted the outcome variable (cognitive/physical fatigue, respectively). Hence, in a regression analysis trait mindfulness was used as a predictor for the criterion variable fatigue. This model yields what is referred to as the *total effect* of mindfulness on fatigue (Fig. 1a, path c). In the second step, the mediator depression was used as a criterion variable and trait mindfulness as a predictor to establish that trait mindfulness was correlated with depression (Fig. 1a, path a). The third step is supposed to demonstrate that the mediator also affects the outcome. Therefore, fatigue was used as the criterion variable, while trait mindfulness and depression were used as predictors in the same regression model (Fig. 1a, path b). In this model, the effect of trait mindfulness on fatigue while controlling for depression is referred to as the *direct effect* (path c´). In the fourth step, it can be tested whether a *complete* or *partial* mediation is present. If depression completely mediated the relationship between trait mindfulness and fatigue, the effect of trait mindfulness on fatigue should be zero, when controlled for depression (*direct effect*, path c´). However, in this context it should be noted that the contemporary literature advises against the term *complete mediation* [40, 41]. Hence, to test whether depression functioned as a significant mediator, a Sobel test [42] was implemented. This test examines whether a potential reduction of the effect of mindfulness on fatigue due to controlling depression as a mediator was significant.

Complementary, the mediation analyses were repeated by means of the PROCESS macro (version 3.5) by Hayes [43] for SPSS (version 22). With this macro, regression coefficients and bootstrap confidence intervals for inference about total, direct and indirect effects may be calculated [41]. In this context, it has been shown that in essence, the basic requirement for a mediation is met if the *indirect effect*, i.e. the product of path a and b (*ab*), is significant. The Sobel test and the implemented macro hence address the same issue. However, in the Sobel test it is assumed that *ab* is normally distributed. Since the sample distribution of *ab* often is not normal, the Sobel test can result in a lower power than tests using bootstrapping (e.g. the PROCESS macro), which do not require a normal sample distribution of *ab* [41].

## Results

Descriptive intercorrelations between trait mindfulness, depression, physical and cognitive fatigue are depicted in Table 2. Trait mindfulness showed a moderate negative correlation with depression and a weak correlation with physical and cognitive fatigue.

**Table 2:** Correlations between trait mindfulness, depression, and fatigue

	Trait Mindfulness	Depression	Physical Fatigue
Trait Mindfulness	–		
Depression	−.637**	–	
Physical Fatigue	−.284*	.661**	–
Cognitive Fatigue	−.299*	.646**	.887**

**Correlation is significant at *p* < .001 (2-tailed)* Correlation is significant at *p* < .05 (2-tailed)

Figure 1 presents a conceptual outline and the main results of the mediation analyses: A significant *total effect* of trait mindfulness on cognitive fatigue was observed (unstandardized B = −.413, *p* = .007, Fig. 1a, path c). After depression was considered as a potential mediator in the model, trait mindfulness predicted depression (path a: B = −.935, *p* < .001, Fig. 1a) and depression predicted cognitive fatigue significantly (B = .723 *p* < .001, Fig. 1a, path b). However, the previously significant effect of mindfulness on cognitive fatigue was reduced and became insignificant (*direct effect:* B = .263, *p* > .05, Fig. 1a, path c´). The Sobel test [42] revealed that the indicated reduction was significant (*z* = − 4.652, *p* < .001), which implies that depression functioned as a significant mediator of the relationship between mindfulness and cognitive fatigue. The same result emerged for the analysis by means of the PROCESS macro [43]; *ab* = −.676, 95% CI [−.877, −.495]. The same steps were performed for the relationship between trait mindfulness and physical fatigue (Fig.1a). Also in this case, both the Sobel test (*z* = − 4.856, *p* < .001) and the analysis by means of the process macro (*ab* = −.697, 95% CI [−.894, −.529]) indicated that depression functioned as a significant mediator of the relationship between mindfulness and fatigue. A graphic illustration of the results of both mediation analyses is provided in Fig.1b. To provide a more comprehensive overview of the results, in Fig.1c and Fig.1d, scatterplots of the relationship between trait mindfulness and depression and between depression and fatigue with the corresponding cut-off scores of the self-report measures are displayed.

## Discussion

The purpose of the current study was to examine the relations between trait mindfulness, depression, and fatigue in PwMS. In particular, the rationale was that mindfulness as an adaptive trait may be negatively associated primarily with depression and less with fatigue. It was hypothesized that fatigue may be regarded as relatively robust, while depression in contrast, might be more susceptible to change by mindfulness. 

In the current study, an exploratory analysis by means of descriptive intercorrelations revealed that trait mindfulness showed a moderate negative correlation with depression and a weak negative correlation with fatigue. In the subsequent mediation analyses, depression mediated the relation between trait mindfulness and fatigue. In particular, after depression was controlled the relationship between trait mindfulness and fatigue became insignificant (Fig. 1a, b). No differences between physical and cognitive fatigue concerning the results of the mediation analyses were evident. 

The results of the current study are generally compatible with the hypothesis that trait mindfulness may alleviate suffering from fatigue by decreasing depressive symptoms. A possible interpretation of these findings might be that depression in MS is more influenced by psychosocial factors [29] such as maladaptive coping than fatigue. Transforming mindfulness from a state to a trait, due to repeated practice of mindfulness and its transfer into daily life, might contribute to a more functional and adaptive coping style [30]. This is in line with previous findings where a nonjudgmental attitude has been associated with less depressive rumination and depression itself [44, 45]. 

While the relation between trait mindfulness and depression has been scrutinized, the connection between depression and fatigue remains less clear. Mohr and colleagues [24], who reported that the reduction of fatigue is primarily associated with an improvement of depressive symptoms following treatment of depression, suggested two explanations for this result: First, it has been shown that individuals high in negative affect tend to complain more about physical symptoms than individuals, who are low in negative affect [46]. Therefore, it might be assumed that depressed PwMS show higher rates of self-reported fatigue than non-depressed patients. A second explanation may be that depression and fatigue share common pathological mechanisms such as inflammatory processes in terms of higher cytokine levels in the central nervous system [25, 28]. It has been shown that treatment of depression may reduce cytokine levels in PwMS [47]. A reduction in depressive symptoms might therefore be accompanied by a decrease in fatigue. 

Previous work focusing on the potential relevance of negative representations and beliefs about fatigue also indicates that beneficial changes in these parameters following cognitive behavioral therapy are associated with reduced fatigue symptoms [48]. As this pattern of results arose independent from changes in mood, it seems likely that the impact of depression on fatigue is not only mediated via changes in mood but also via changes in thought patterns. In sum, the findings of the current study confirm and complement the findings from previous studies and suggest that subjective suffering from fatigue may be strongly affected by symptoms of depression, including negative mood, as well as maladaptive and dysfunctional attitudes about fatigue. 

A clear limitation of the current work is that it used a cross-sectional design and that the basic assumptions underlying the study rationale may be regarded as somewhat simplified. Fatigue is not just a direct consequence of MS-related physiological changes and depression not just due to psychosocial problems. Psychosocial factors are also relevant for fatigue and neurobiological processes play an important part in depression [28, 31, 48]. Yet, based on the concurrent literature, it seems appropriate to suggest that psychosocial factors play a more prominent role in the etiology of depression compared to fatigue in MS [25–27, 29]. 

Another limitation of the current study is that no measures of pain were included. Many PwMS suffer from pain, which has been closely associated with both fatigue [31] and depression [49]. It has even been suggested that pain, fatigue and depression represent a symptom cluster in PwMS and that treating one of these symptoms may affect the others [50, 51]. 

Finally, a considerable part of the current sample showed only mild symptoms of depression and fatigue. This might be related to the relatively low neurological impairment in the sample, which is represented by the low EDSS level and the fact that all patients had a diagnosis of RRMS. However, since mindfulness training has also been shown to be effective in patients with residual symptoms and clinically relevant depressive symptoms [52, 53], one might assume that the results can also be generalized to a patient population with more pronounced depressive symptoms. 

Within the limitations outlined above, the findings of the current study are relevant for clinical studies in MS in which MBIs are applied. Several studies in this field reported that MBIs are effective in relieving symptoms of fatigue [13–15, 23]. The current study provides complementary information in this context. The results suggest that mindfulness might be particularly relevant to counteract depression in MS which in turn can reduce fatigue. Hence, it may be suggested that the central target of MBIs should be the reduction of depressive symptoms and not of fatigue per se. Trait mindfulness might reduce depression which in turn might help to effectively cope with the psychological stress caused by fatigue. 

For those PwMS who suffer predominantly from fatigue and not from depressive symptoms it remains to be examined, whether mindfulness can be beneficial. Previous work has shown that it is possible to identify this subgroup of patients [54]. Therefore, further research on the feasibility and effectiveness of treatment approaches for ameliorating fatigue in the absence of depressive symptoms is warranted. The role of pain as a potential factor in the constellation of trait mindfulness, depression and fatigue, as well as patients with other MS subtypes (SPMS and PPMS), should be considered in this context.

## Data Availability

The data that support the findings of this study are available on request from the corresponding author, ToS.
